# Primary Plasma Cell Leukemia: Recent Advances in Molecular Understanding and Treatment Approaches

**DOI:** 10.3390/ijms26136166

**Published:** 2025-06-26

**Authors:** Ichiro Hanamura, Sivasundaram Karnan, Akinobu Ota, Akiyoshi Takami

**Affiliations:** 1 Division of Hematology, Department of Internal Medicine, Aichi Medical University, Nagakute 480-1195, Japan; takami-knz@umin.ac.jp; 2 Department of Biochemistry, Aichi Medical University, Nagakute 480-1195, Japan; skarnan@aichi-med-u.ac.jp; 3 Department of Food and Nutritional Environment, College of Human Life and Environment, Kinjo Gakuin University, Nagoya 463-8521, Japan; aota@kinjo-u.ac.jp

**Keywords:** plasma cell leukemia, circulating plasma cells, t(11;14), venetoclax, CAR-T, bispecific antibodies

## Abstract

Primary plasma cell leukemia (pPCL) is a rare and aggressive plasma cell dyscrasia. According to revised diagnostic criteria, pPCL is defined by the presence of ≥5% circulating plasma cells (CPCs) in the peripheral blood of patients with newly diagnosed multiple myeloma (NDMM). pPCL is characterized by a distinct cytogenetic profile, including frequent t(11;14), *MAF/MAB* translocations, 1q gain, and del(17p). While t(11;14) is generally associated with a favorable prognosis, the coexistence of multiple high-risk cytogenetic abnormalities is linked to poorer outcomes. Tandem autologous hematopoietic stem cell transplantation and novel anti-myeloma agents have improved survival in some patients; however, overall prognosis remains poor, particularly in those ineligible for transplantation. Venetoclax and emerging immunotherapies, such as CAR-T cells and bispecific antibodies, show promise and merit clinical trials focused on pPCL-enriched cohorts. Additionally, recent findings associating even minimal CPCs with adverse outcomes in NDMM support broader inclusion criteria in future trials. A deeper understanding of pPCL’s molecular pathology is critical for the development of effective targeted therapies. This article reviews recent advances in the molecular understanding of and treatment strategies for pPCL.

## 1. Introduction

Plasma cell leukemia (PCL), which is defined as the presence of plasma cells in peripheral blood [1,2], is classified as primary PCL (pPCL), which occurs in patients with newly diagnosed multiple myeloma (NDMM), and secondary PCL, which develops in those with relapsed refractory MM (RRMM) [3,4]. PCL suggests the presence of MM cells with a growth advantage, which can survive without the support of the bone marrow microenvironment. Studies have shown that even low levels of circulating plasma cells (CPCs), defined as <5%, are associated with poor prognosis in patients with NDMM [5,6,7,8,9].

pPCL is very rare, accounting for approximately 1–2% of NDMM cases [10,11]. pPCL progresses rapidly and can lead to early death if left untreated; therefore, immediate diagnosis and treatment are critical in patients with pPCL. The clinical and cytogenetic features of pPCL are distinct from those of NDMM [1,2,12,13,14]. The clinical outcomes of pPCL remain unfavorable despite the introduction of novel anti-MM agents [11,15,16]. However, given its rarity, pPCL is usually excluded from prospective randomized clinical trials for patients with NDMM and the establishment of standard optimal therapeutic regimens has been challenging. Emerging immunotherapeutics—such as chimeric antigen receptor T cells (CAR-Ts), bispecific antibodies (BsAbs), and antibody–drug conjugates (ADCs) targeting B-cell maturation antigen (BCMA) and other markers—have shown high efficacy in patients with heavily pretreated MM [17,18], and are expected to offer therapeutic benefit in pPCL as well. In this review, we summarize the recent advances in the diagnosis, molecular understanding and treatment approaches of pPCL.

## 2. Diagnostic Criteria and Clinical Features of pPCL

**Figure 1:**
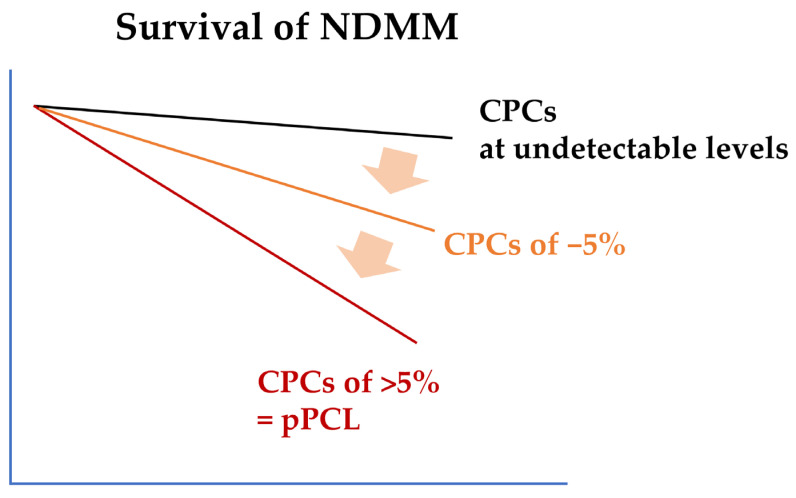
**A schematic diagram showing the relationship between CPC levels and overall survival in NDMM.** In NDMM, even low CPC levels are a poor prognostic factor. CPC is a continuous variable, and survival rates decrease as the proportion of CPCs increases. Currently, patients with CPCs of 5% or more are defined as pPCL. CPC, circulating plasma cell; NDMM, newly diagnosed multiple myeloma; pPCL, primary plasma cell leukemia.

Before the revised diagnostic criteria were updated in 2021 [4], the initial diagnostic criteria for pPCL, which were proposed by Kyle et al. in the 1970s, required the presence of ≥20% CPCs with an absolute CPC count of ≥2 × 10^9^/L in the peripheral blood [1]. In the 2010s, following the development of novel agents for MM, studies reported that patients with a CPC level of 5–19% exhibited poor overall survival (OS) comparable to those observed in patients with ≥20% CPCs [19,20]. The newly proposed criteria for the diagnosis of pPCL, published by the International Myeloma Working Group in 2021, include a lower percentage of CPCs (≥5%) as a threshold [4]. Subsequently, several studies have confirmed that the revised diagnostic criteria are appropriate for the diagnosis of pPCL, in terms of prognostic value [21,22]. However, with more improvements in anti-myeloma treatment, several recent studies have reported that even a CPC level of <5% is an independent poor prognostic factor for NDMM (Figure 1) [5,6,9]. The current diagnostic criterion for pPCL, a cutoff value of 5%, is arbitrary, and recent studies have demonstrated the prognostic value of CPCs as a continuous variable [8,23]. More suitable or nuanced diagnostic criteria that better reflect the biological and clinical spectrum of NDMM with CPCs are required.

CPCs harboring t(11;14) often exhibit a lymphocyte-like appearance in smears and may be difficult to morphologically distinguish; therefore, flow cytometry is recommended for the detection of CPCs [24].

The incidence of pPCL is very low, with an estimated rate of 1% in patients with NDMM [25,26]. The median age at pPCL diagnosis is approximately 60 years, which is younger than that of the patients with NDMM. Severe anemia, thrombocytopenia with platelet counts of <100,000/L, renal insufficiency, hypercalcemia, high lactate dehydrogenase levels, advanced International Staging System stage, and extramedullary disease are more common in patients with pPCL [25,27,28]. Compared with typical NDMM, pPCL progresses rapidly and can lead to early death, requiring immediate diagnosis and intervention with antitumor therapeutics. In patients with pPCL, positron emission tomography/computed tomography (PET/CT) is recommended for the detection of extramedullary lesions, an important cause of early death [29,30].

## 3. Biologic Features of pPCL

**Figure 2:**
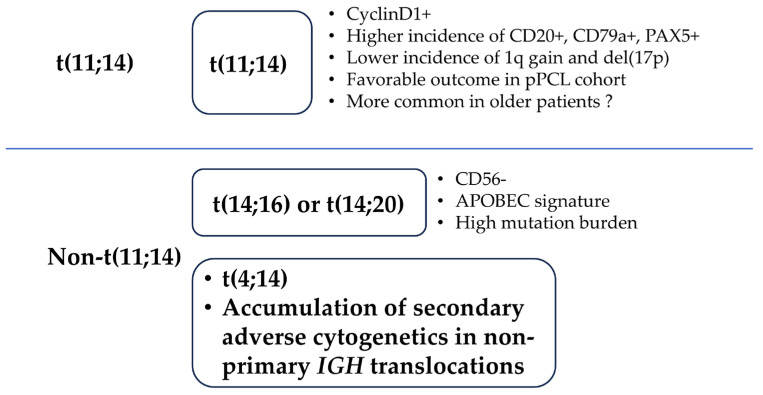
**A schematic diagram of the proposed molecular pathology of pPCL based on chromosomal abnormalities.** pPCL is classified into two types: one type that develops based on t(11;14) and another type that develops based on other factors. pPCL: primary plasma cell leukemia; PAX5: paired box protein 5; APOBEC: apolipoprotein B mRNA editing enzyme, catalytic polypeptide-like.

Although pPCL shares genomic abnormalities with MM, the genetic profile, including the cytogenetics, of pPCL is distinct from that of NDMM [3,14]. The incidence of t(11;14), t(14;16), t(14;20), del(1p), del(13q), del(17p), 1q gain, and hypodiploidy are higher in patients with pPCL than in those with NDMM [12,13,27,31,32]. Except for the higher frequency of t(11;14), pPCL has similar cytogenetic features to those of high-risk NDMM (i.e., a high frequency of high-risk cytogenetics and multiple high-risk cytogenetics) [33]. Recently, van de Donk et al. reported that the cytogenetic profile differed between the patients aged ≤65 years and those aged >66 years among the patients with pPCL enrolled in the EMN12/HOVON-129 study [34]. The authors reported that the frequency of t(11;14) was lower whereas the frequencies of t(14;16) and del(17p) were higher in patients with pPCL aged ≤65 years (Table 1) [34]. These findings might suggest that patients are categorized into those who develop pPCL in association with t(11;14), which is more commonly observed in older individuals, and those who develop pPCL in association with high *MAF/MAFB* expression and/or poor prognostic secondary abnormalities, which are more commonly observed in younger individuals (Figure 2).

Whole-exome sequencing has revealed that patients with pPCL exhibit a highly heterogeneous mutational profile [35]. The number of nonsynonymous mutations is higher in pPCL than in NDMM, which is potentially due to a greater prevalence of the APOBEC mutational signature induced by *MAF/MAFB* translocations [35]. *TP53* mutations, including the bi-allelic inactivation of *TP53*, are more common in patients with pPCL than in those with NDMM [32,35].

In pPCL, malignant plasma cells typically express plasma cell markers, including CD38 and CD138. Compared to NDMM, pPCL cells more frequently express CD19, CD20, CD27, CD44, and CD45, and less frequently express CD56, CD117, and human leukocyte antigen-DR (HLA-DR) [12,36]. The infrequent expression of CD56 may correlate with the higher incidence of *MAF/MAFB* translocations in pPCL. The downregulation of adhesion molecules, such as CD44 and CD56, can disrupt the adhesion of plasma cells to the bone marrow stroma, facilitating their spread to extramedullary sites, including the peripheral blood. The lack of CD27 expression may be linked to nuclear factor kappa B-mediated antiapoptotic pathways [37]. The loss of HLA-DR expression in pPCL may reflect immune evasion by the tumor. HLA-DR is essential for presenting antigens to CD4^+^ T cells, and its absence impairs immune recognition, which might contribute to the aggressive behavior and rapid progression of pPCL.

Paiva et al. reported differences in the immune phenotypes of CPCs and myeloma cells in the bone marrow of the same patients [38]. CPCs constituted a unique subpopulation of clonal myeloma cells in the bone marrow, characterized by the downregulation of integrins (CD11a/CD11c/CD29/CD49d/CD49e), adhesion (CD33/CD56/CD117/CD138), and activation molecules (CD28/CD38/CD81). In addition, this paper reported that CPCs are quiescent but clonogenic and, interestingly, exhibit a fluctuating circadian rhythm.

The gene expression profile of pPCL is also distinct from that of NDMM, including the higher expression of *TAGLN2*, *RUNX4*, *PHF19*, *DEPTOR*, *FAS*, *FAM72D*, and *TOX2* and the lower expression of *VCAM1*, *CXCL12*, *PPBP, CD163*, and *DKK1* [32,35]. Hofste Op Bruinink et al. reported that pPCL was identified on the basis of a specific tumor transcriptome signature, which was also present in patients with high-risk NDMM, who constitute around 10% of patients, despite not being clinically leukemic [39]. Such patients might be in the pre-stage of pPCL.

pPCL patients with t(11;14) exhibit a lower incidence of 1q gain and del(17p), and higher expression of immature B-cell markers such as PAX5 and CD79A, compared to those without t(11;14), suggesting distinct oncogenic mechanisms [32,40]. In a study of patients with NDMM and CPC levels of 0.105–5%, Xia et al. reported that those with mutations in *TP53, BRAF, DNMT3A,* and *TENT5C*, and the genes related to the interleukin 6/Janus kinase/signal transducer and the activator of the transcription 3 (IL-6/JAK/STAT) pathway tended to have higher levels of CPCs [41]. This suggests that pPCL cells can proliferate in an IL-6-independent manner, aided by mutations that promote cell survival and growth.

Patients with pPCL often present with disseminated extramedullary lesions (EMDs) [30,42], which appear to be caused by the hematogenous metastasis of MM cells. On the other hand, some NDMM patients have tumorous EMDs without clear leukemic status. Compared with typical NDMM, both pPCL and NDMM with tumorous EMD show a poor prognosis [42,43], but pPCL generally shows a poorer prognosis and is more aggressive, with a higher tumor burden and more rapid progression than NDMM with tumorous EMDs. This may be related to the higher chromosomal instability and higher mutation load in pPCL, which result in more aggressive tumors. Garcés et al. reported that CPCs were detected in the blood of all MM patients using advanced detection techniques (next-generation flow, NGF) (n = 53), showing high concordance with bone marrow myeloma cells in copy number changes at chromosomal arm levels (>95%) but low in translocations (39%). High-risk cytogenetic abnormalities and >82% of mutations were also captured. About 22% of CPCs originated from different marrow or extramedullary sites, indicating spatial heterogeneity [43].

Del(17p) is known to be frequently observed both in pPCL and tumorous EMD, but molecular abnormalities specific to pPCL that are not observed in tumorous EMD are not well characterized. Rojas et al. performed transcriptome analysis of pPCL and NDMM, both of which have del(17p), and reported that the abnormal expression of spliceosome components was significantly observed in pPCL with del(17p) compared to NDMM with del(17p). This suggests that the intracellular mRNA processing pathway including RNA splicing machinery is different between pPCL with del(17p) and NDMM with del(17p) [44].

The clinical features and molecular pathology of pPCL overlap those of ultrahigh-risk NDMM, but the frequency of patients with t(11;14), which is a favorable marker in NDMM, is clearly higher in pPCL [33,45]. This suggests once again that pPCL could be classified into two types: one that develops in association with t(11;14) and another that is a leukemic type of ultrahigh-risk NDMM (Figure 2).

Research on the molecular pathology of pPCL supports the development of targeted therapies. As pPCL is an aggressive tumor with highly complex and multifaced molecular abnormalities, targeted drugs may be more effective when used in combination with other anti-myeloma agents rather than as monotherapy. In the future, progress in basic research directly related to pPCL treatment is expected.

## 4. Treatment and Perspectives

**Figure 3:**
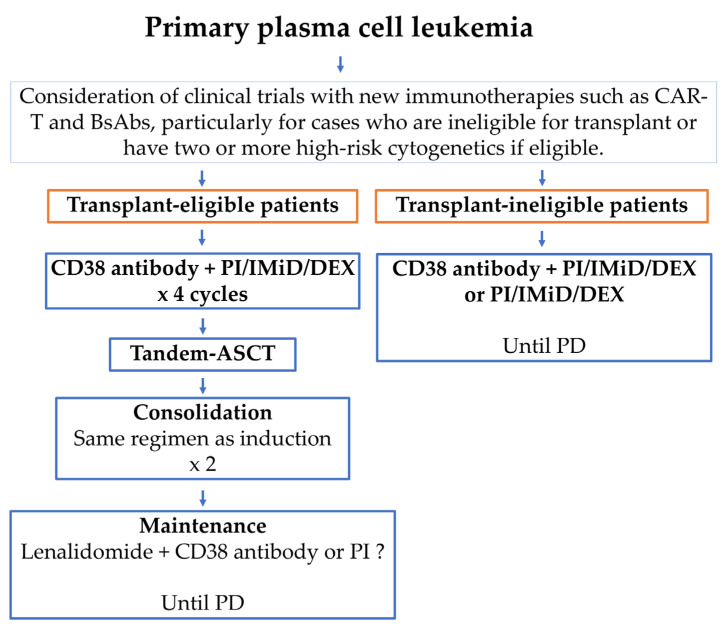
**Treatment approach for patients with newly diagnosed pPCL.** CAR-T, chimeric antigen receptor T cell; BsAb, bispecific antibody; PI, proteasome inhibitor; IMiD, immunomodulatory drug; DEX, dexamethasone; ASCT, autologous hematopoietic stem cell transplantation; PD, progressive disease.

Historically, pPCL has been associated with high early mortality and extremely poor OS. Conventional MM chemotherapy in pPCL has shown a response rate of less than 50% and a 5-year OS of less than 10% [46]. Novel anti-MM agents—such as CD38 antibodies, proteasome inhibitors (PIs), and immunomodulatory drugs (IMiDs)—have improved response rates, the quality of response, progression-free survival (PFS), and OS in pPCL compared to conventional chemotherapy, although not to the extent seen in NDMM [7,22,47,48,49]. Currently, CD38 antibody-based quadruplet regimens—including a CD38 antibody, PI, IMiD, and dexamethasone (DEX)—are recommended as the optimal frontline therapy for patients with pPCL (Figure 3) [22,30]. Autologous hematopoietic stem cell transplantation (ASCT), especially tandem ASCT followed by consolidation and maintenance therapy, has been shown to reduce early relapse and improve survival, and is therefore recommended for eligible patients [3]. There is no established post-transplant treatment regimen for CD38 antibody-based quadruple therapy; however, since treatment failure in pPCL can lead directly to death, consolidation therapy is recommended to be the same as the induction therapy that has been demonstrated to be effective [30]. This will help deepen the response and reduce the risk of early recurrence. Maintenance therapy will be continued for a long period of time until progression or intolerance, and will consist of a two-drug combination therapy based on lenalidomide excluding steroids [30].

Tandem ASCT may be particularly beneficial for pPCL patients who achieve complete remission (CR) before transplantation [50,51]. Regarding allogeneic SCT (allo-SCT), myeloablative conditioning allo-SCT has resulted in long-term survival with a plateau phase in approximately 20% of patients; however, this approach carries a high risk of treatment-related mortality (TRM) [52]. In contrast, reduced-intensity conditioning (RIC) allo-SCT is associated with lower TRM but a higher relapse rate in pPCL [52]. Tandem ASCT/RIC-allo-SCT may be an option when a graft-versus-myeloma effect is desired [52]. The disease status prior to first transplantation may guide the choice of transplant modality: patients achieving CR may benefit from tandem ASCT, while those not achieving CR may benefit more from tandem ASCT/RIC-allo-SCT [51]. The role of SCT in the era of CD38 antibody-based quadruplets and novel immunotherapies (including CAR-T, BsAbs and ADCs) has not been fully defined in pPCL. Although transplantation can improve survival, many pPCL patients are ineligible due to age, comorbidities, or delayed diagnosis and ineffective initial treatment—even among younger patients. Therefore, early detection and effective initial therapy are critical to enabling transplantation and improving outcomes.

As in NDMM, achieving minimal residual disease (MRD) negativity—rather than merely CR—may be a more meaningful prognostic indicator in pPCL [6,9,34]. In the EMN12/HOVON-129 study, 28% of younger patients (10 out of 36) with pPCL (CPCs ≥ 20%) achieved MRD negativity [34]. These MRD-negative patients were overrepresented among long-term survivors and had superior outcomes. This highlights MRD negativity as a strong prognostic indicator even in high-risk diseases like pPCL. In addition, it has been reported that achieving MRD negativity improved PFS in transplant-eligible NDMM patients at any level of CPCs detectable by flow cytometry [6,7]. Sustained MRD negativity may serve as a strong surrogate marker, as pPCL cells tend to proliferate more rapidly than those of typical NDMM. The optimal timing for MRD assessment in pPCL has not been established, but considering the aggressiveness of pPCL, it is recommended to perform MRD assessment multiple times after induction therapy, post-ASCT, after consolidation therapy, and during maintenance therapy. In pPCL, MRD positivity may be associated with early clinical relapse, and therefore it is recommended to start treatment as early as possible. In particular, in cases with MRD positivity after ASCT, the induction or consolidation therapy may have been insufficient. Therefore, it may be appropriate to promptly initiate salvage treatment with CAR-T cells or bispecific antibodies, which have distinct mechanisms of action from the prior therapy.

Due to its rarity and frequent exclusion from large NDMM trials, most available data on pPCL treatment outcomes are derived from small retrospective studies. To date, only three prospective phase II trials have been conducted for newly diagnosed pPCL (all patients had ≥20% CPCs) (Table 2) [46]. The Gruppo Italiano Malattle Ematologiche d’Adulto (GIMEMA) trial investigated lenalidomide and dexamethasone in 23 patients with pPCL, including 15 transplant-eligible (TE) and 8 transplant-ineligible (TIE) patients [46]. With a median follow-up of 34 months, the median PFS and OS were 27 months and not-reached, respectively, in TE patients and 2 and 12 months, respectively, in TIE patients. Among the 15 TE patients, 9 underwent SCT (single ASCT [n = 4], double ASCT [n = 4], ASCT/RIC-allo [n = 1]). Survival was better among those who received transplantation.

The Intergroupe Francophone du Myélome (IFM) trial investigated the efficacy of bortezomib-based induction therapy followed by SCT in patients with newly diagnosed pPCL. The treatment regimen included four alternating cycles of PAD (bortezomib + doxorubicin + dexamethasone) and VCD (bortezomib + cyclophosphamide + dexamethasone), followed by ASCT. Eligible patients then received RIC-allo-SCT (=tandem auto/RIC-allo), while the remaining patients received a second ASCT (=tandem auto/auto) along with consolidation therapy using VRD (bortezomib + lenalidomide + dexamethasone) and one year of lenalidomide maintenance [53]. Forty transplant-eligible (TE) patients (aged 70 years or younger) were enrolled. One patient died from septic shock before the start of induction therapy, and 35 patients completed the planned four cycles of PAD/VCD induction. Following induction, 27 patients (69%) achieved stable disease or better, including 14 patients (36%) who achieved VGPR or better, and 25 patients proceeded to the first ASCT. Of the 23 patients who achieved VGPR or better after the first ASCT, 16 patients (70%)—all younger than 66 years and with an available donor—underwent RIC-allo-SCT (=tandem auto/RIC-allo), while the remaining 7 underwent a second ASCT (=tandem auto/auto) followed by post-transplant therapy. With a median follow-up of 28 months, the median OS and PFS were 36 and 15 months, respectively, in the overall cohort. From the date of the second SCT, the median OS and PFS were 36 and 17 months, respectively, in the RIC-allo-SCT group (=tandem auto/RIC-allo), while the median OS and PFS were not reached in the ASCT plus post-transplant therapy group (=tandem auto/auto). These results indicate that bortezomib-based induction followed by SCT provides a high response rate and improved PFS compared to historical cohorts, and that tandem auto/auto with VRD/lenalidomide may offer superior outcomes over tandem auto/RIC-allo.

The EMN12/HOVON-129 trial included 61 patients with pPCL who were treated with a regimen including carfilzomib and lenalidomide in combination with SCT, if eligible [34]. Patients aged 18–65 years (younger patients) and those aged 66 years or older (older patients) were treated in age-specific cohorts. Patients aged 65 years or younger received four cycles of induction therapy with carfilzomib, lenalidomide, and dexamethasone (KRd), followed by either tandem ASCT (=tandem auto/auto) or ASCT/RIC-allo-SCT (=tandem auto/RIC-allo). Patients in the tandem ASCT group received four cycles of consolidation therapy with KRd and maintenance therapy with KR until disease progression or unacceptable toxicity. Patients eligible for allo-SCT with an available donor underwent a single RIC-allo-SCT (=tandem auto/RIC-allo), followed by carfilzomib maintenance and KR until progression or undue toxicity. Patients aged 66 years or older received eight cycles of KRd induction therapy, followed by KR maintenance. In the younger cohort, the ORR, ≥VGPR, and CR rates were 86%, 83%, and 50%, respectively. Among the 20 evaluable patients, 16 (80%) achieved MRD negativity. At a median follow-up of 43 months, the median PFS and OS were 15 and 28 months, respectively; outcomes were comparable between the tandem ASCT (=tandem auto/auto) and ASCT/RIC-allo-SCT (=tandem auto/RIC-allo) groups. In the older cohort, the ORR, ≥VGPR, and CR rates were 80%, 68%, and 36%, respectively. Among the eight evaluable patients, five (63%) achieved MRD negativity. With a median follow-up of 32 months, the median PFS and OS were 13 and 24 months, respectively—results superior to those previously reported in transplant-ineligible patients. These three studies suggest that novel anti-myeloma drugs (particularly PI/IMiD/DEX triplets), followed by tandem ASCT with consolidation and maintenance therapy, are more likely to improve outcomes in pPCL. However, the overall prognosis of pPCL remains unsatisfactory compared to that of NDMM.

The rapidly evolving treatment paradigm for MM is expected to drive advances in the management of pPCL. CAR-T therapy and BsAbs therapies have shown high efficacy in heavily treated refractory or relapsed MM, and are promising therapeutic options for pPCL [54]. In daily clinical practice, these therapies have already been shown to be highly effective in refractory or relapsed pPCL. Several case studies have reported the efficacy of CAR-T and BsAbs in refractory or relapsed pPCL [55,56]. Ongoing phase II clinical trials are currently evaluating the efficacy and safety of BCMA-targeted CAR-T therapy as a front-line treatment for newly diagnosed pPCL (NCT05979363 and NCT05870917) (Table 3). Xu et al. reported at ASH 2024 that all four patients who completed the full “sandwich” regimen—comprising CAR-T1/ASCT/CAR-T2/R-maintenance—for transplant-eligible, newly diagnosed pPCL achieved MRD-negative complete responses (NCT05870917) [57]. Currently, there are no clinical trials evaluating BsAbs in the front-line setting for pPCL. In CAR-T cell therapy, the interval from apheresis to infusion—referred to as the manufacturing turnaround time—is typically around one month. Given that some pPCL patients are refractory to induction therapy, the earlier use of BsAbs is also being considered. CT071, an anti-GPRC5D CAR-T product with a turnaround time of as little as two days from apheresis to infusion, represents an attractive treatment option for patients with rapidly progressing pPCL (NCT05838131) (Table 3). As pPCL has a high tumor burden and is an extramedullary disease itself, therapies with CAR-T and BsAbs may not improve outcomes as much as they do for MM. However, when used as first-line therapy, these therapies may avoid T-cell exhaustion, immune dysfunction, and a highly immunosuppressive tumor microenvironment, and are therefore expected to be more effective than anticipated. pPCL with t(11;14) has a lower frequency of high-risk cytogenetic abnormalities of NDMM, and is expected to have lower intrinsic tumor aggressiveness, resulting in novel immunotherapies potentially being more effective in these cases.

Venetoclax, a BCL-2 inhibitor, also shows promise in pPCL with t(11;14) [32]. BCL-2 is an anti-apoptotic protein that promotes cancer cell survival, and myeloma cells with t(11;14) are particularly dependent on BCL-2. Venetoclax blocks BCL-2, activates the apoptotic pathway, and selectively eliminates BCL-2-dependent tumor cells. As a result, venetoclax is especially effective in t(11;14)-positive multiple myeloma, often inducing deep and durable responses [58,59]. Venetoclax, either as monotherapy or in combination with daratumumab and other agents, may be a viable treatment option for this subset of pPCL patients [60,61].

Although long-term survival has been achieved in a subset of patients treated with novel anti-MM agents in combination with tandem ASCT followed by continuous post-transplant therapy, the overall prognosis of pPCL remains poor. There is an urgent need to improve outcomes, particularly for patients without t(11;14), with ≥2 high-risk cytogenetic abnormalities, and those who are ineligible for transplantation. Clinical trials evaluating new therapeutics, especially emerging immunotherapies, are essential for improving the prognosis of pPCL.

In summary, future clinical trials should explore the earlier-line integration of CAR-T cells and BsAbs into the CD38-based quadruplet and tandem ASCT strategy for pPCL. Venetoclax trials are also warranted for patients with t(11;14). These studies will help identify novel prognostic markers, support risk-adapted treatment approaches, and guide the development of targeted therapies. MRD-guided strategies for treatment escalation or de-escalation may further advance pPCL management.

## 5. Conclusions

A rare plasma cell dyscrasia, pPCL is characterized by a distinct genetic profile, rapid progression, and resistance to conventional MM chemotherapies, with a limited response to novel anti-MM drugs compared to NDMM. In pPCL, t(11;14), *MAF/MAFB* translocations, and the disruption of *TP53* are more frequently observed. Patients with t(11;14) and those without it exhibit different molecular features, outcomes, and age distributions. Therefore, it may be possible to develop a new classification system based on the presence or absence of t(11;14) and to establish new treatment strategies, including the use of venetoclax. In pPCL, due to its refractory and aggressive nature, continuous intensified treatment is essential for long-term survival beginning immediately after diagnosis. Currently, CD38-based quadruplet therapy followed by tandem ASCT with consolidation and maintenance therapy is recommended for eligible patients and has provided long-term survival in a subset of the pPCL population. However, many patients with pPCL are not eligible for transplantation due to delayed diagnosis, the inadequate efficacy of initial therapy, age, and frailty. Therefore, early diagnostic systems (e.g., the establishment of regional referral systems and the detection of CPCs using flow cytometry in patients with NDMM) and the development of well-tolerated and effective treatments are important to improve outcomes in pPCL. There is an urgent need for clinical trials to evaluate the efficacy and safety of CAR-T, BsAbs, ADCs, and venetoclax in patients with pPCL. In addition, the further elucidation of the molecular pathology of pPCL and the development of targeted drugs that can reduce or overcome drug resistance will support the advancement of effective treatment strategies in the future.

## Figures and Tables

**Table 1:** Cytogenetic and clinical characteristics in primary plasma cell leukemia at diagnosis.

Cytogenetic Abnormality	EMN12/HOVON129 n = 61, %			IFM n = 32, %	GIMEMA n = 23, %
Age, years	Overall (52–74)	52–63 (n = 36)	69–74 (n = 25)	70 years old and younger	44–80
t(4;14)	6	9	4	6	13
t(14;16)	11	19	4	16	30
t(14;20)	N/A	N/A	N/A	N/A	4
t(11;14)	32	26	55	50	30
Hyperdiploidy	8	13	17	9	N/A
1q ≥ 3 copies	51	61	52	53	43
del(1p)	26	36	0	16 (homozygous deletion of *CDKN2C*)	N/A
del(13q)	54	71	52	59	70
del(17p)	31	47	18	28	30
del(17p), t(4;14), or t(14;16)	40	59	27	N/A	N/A
≥2 high-risk cytogenetics (del(17p), t(4;14), or t(14;16))	24	34	14	N/A	38 (del(17p), t(4;14), t(14;16), t(14;20), 1q gain)
Clinical characteristics					
IgG	40	31	56	37	N/A
IgA	11	19	0	12	N/A
IgM	1	0	4	2	N/A
IgD	6	6	8	5	N/A
Light chain only	39	44	32	32	N/A
EMD other than PB	14	17	12	12	8
ISS stage 2	21	25	17	43	17
ISS stage 3	65	64	74	43	61

IFM, Intergroupe Francophone du Mye′ lome; GIMEMA, Gruppo Italiano Malattle Ematologiche d’Adulto; EMD, extramedullary disease; PB, peripheral blood; ISS, International Staging System.

**Table 2:** Summary of phase II prospective trials for primary plasma cell leukemia.

Study Name or Group (Years)	Patients	Treatments	Actual Transplant (s) (Number)	Results	
				**Response, %**	**Median PFS/OS (months)**
**GIMEMA** [46] **(2009–2011)**	N = 23 TE 15, TIE 8	**Induction** Lenalidomide + dexamethasone	Single ASCT 4 Double ASCT 4 ASCT/RIC-allo 1	**Overall** ORR 73 (CR 13, VGPR 26)	**Transplant-eligible (n = 15)** PFS/OS 21/not reached **Transplant-ineligible (n = 8)** PFS/OS 10/12
**IFM** [53] **(2010–2013)**	N = 40 All patients were TE Median age 57 years (range, 27 to 71)	**Induction** Bortezomib + dexamethasone + doxorubicin/cyclophosphamide (PAD/VCd)	ASCT/RIC-allo 16 ASCT/ASCT 9 (One patient received a syngeneic transplant conditioned with high-dose melphalan.)	**Overall** ORR 69 (CR 10, VGPR 26) **After induction (n = 39)** ORR 69 (sCR 0, CR 10, VGPR 26, PR 23) Refractory disease 25, early death 5 in induction **After 1st ASCT (n = 26)** ORR 92 (sCR 4, CR 34, VGPR 38, PR 16, PD 8)	**Overall** PFS/OS 15/36 **ASCT/ASCT (n = 7)** (From the second transplant) PFS/OS not reached/not reached **ASCT/RIC-allo (n = 17)** (From the second transplant) PFS/OS 11/28
**EMN12/HOVON129** [34] **(2015–2021)**	N = 61 TE 36 TIE 25	**TE (n = 36)** **Induction** 4 cycles of carfilzomib + lenalidomide + dexamethasone (KRd) **Consolidation** 4 cycles of KRd in ASCT/ASCT **Maintenance** KR in ASCT/ASCT and ASCT/RIC-allo (until PD)	1st ASCT 24 ASCT/ASCT 12 ASCT/RIC-allo 5	**After induction (n = 36)** ORR 83 (sCR 3, CR 11, VGPR 61, PR 8, PD 8) **After 1st ASCT (n = 24)** ORR 96 (sCR 13, CR 21, VGPR 63, PD 1) **After 2nd ASCT (n = 12)** ORR 100 (sCR 8, CR 17, VGPR 75) **After RIC-allo (n = 5)** ORR 100 (sCR 40, CR 40, VGPR 20)	**Overall cohort of TE** PFS/OS 15/28 (Early mortality rate 8% at 6 months) **After 1st ASCT (n = 24)** PFS/OS 26/not reached (From date of first ASCT) **After 2nd ASCT (n = 12)** PFS/OS at 2 years 58%/82% (From the second transplant) **After RIC-allo (n = 5)** PFS/OS at 2 years 60%/53% (From the second transplant)
		**TIE (n = 25)** **Induction** 8 cycles of carfilzomib + lenalidomide + dexamethasone (KRd) **Maintenance** KR until PD	None	**After induction cycle 1–4 (n = 25)** ORR 80 (sCR 12, CR 12, VGPR 44, PR 12); 2 early death, 2 excessive toxicity **After induction cycle 5–8 (n = 19)** ORR 95 (sCR 21, CR 21, VGPR 47, PR 5, PD 1)	PFS/OS 13/24 (Early mortality rate 16% at 6 months)

GIMEMA, Gruppo Italiano Malattle Ematologiche d’Adulto; IFM, Intergroupe Francophone du Mye′ lome; pPCL, primary plasma cell leukemia; TE, transplant-eligible; TIE, transplant-ineligible; ASCT, autologous hematopoietic stem cell transplantation; RIC, reduced intensity conditioning; allo, allogeneic hematopoietic stem cell transplantation; PD, progressive disease; ORR, overall response rate; sCR, stringent complete remission; CR, complete remission; VGPR, very good partial remission; PR, partial remission; OS, overall survival; PFS, progression-free survival.

**Table 3:** Selected clinical trials of CAR-T for pPCL.

NCT Number	Study Title	Candidates and Targets of CAR-T	Study Phase and Treatment Flow	Brief Summary	Comments
**NCT05979363**	A Study of Bortezomib, Lenalidomide and Dexamethasone (VRd) Followed by BCMA CAR-T Therapy in Transplant-Ineligible Patients With Primary Plasma Cell Leukemia	Newly diagnosed pPCL Anti-BCMA CAR-T Cells	Phase 2 VRd x3→CAR-T →VR x3 →VR maintenance	A single-arm, open-label study to evaluate the efficacy and safety of VRD-based regimen combined with BCMA CAR-T in transplant-ineligible patients with primary plasma cell leukemia.	CART without ASCT in patients (age ≤ 75 years old).
**NCT05870917**	A Study of VRD-based Regimen Combined With CART-ASCT-CART2 Treatment in Patients With Primary Plasma Cell Leukemia	Newly diagnosed pPCL Anti-BCMA CAR-T Cells	Phase 2 VRd x3→1st CAR-T →VR x3→2nd CAR-T →R maintenance	A single-arm, open-label study to evaluate the efficacy and safety of a VRD-based regimen (Bortezomib, Lenalidomide, and Dexamethasone) combined with CART-ASCT-CART2 in Chinese patients with newly diagnosed primary plasma cell leukemia.	CART-ASCT-CART2 sandwich regimen in transplant-eligible patients (age ≤ 65 years old). [57]
**NCT05838131**	A Clinical Trial to Explore the Safety and Efficacy of CT071 Injection in Patients With Relapsed/Refractory Multiple Myeloma or Primary Plasma Cell Leukemia	Relapsed/refractory MM and relapsed/refractory pPCL Anti-GPRC5D CAR-T Cells (CT071)	Phase 1/2 study	A single-arm, open-label, dose-finding, first-in-human clinical trial. The main aim of this study is to preliminarily evaluate the safety and tolerability of CT071 after infusion, and explore the dose range of CT071 in patients with relapsed/refractory multiple myeloma or primary plasma cell leukemia, so as to determine the possible recommended therapeutic dose (RD).	Shortened manufacturing process to around 30 h in vein-to-vein time.

pPCL, primary plasma cell leukemia; CAR-T, chimeric antigen receptor—T cell; MM, multiple myeloma; BCMA, B-cell maturation antigen; GPRC5D, G protein-coupled receptor, class C, group 5 member D.

## Data Availability

Not applicable.

## Abbreviations

The following abbreviations are used in this manuscript:

ADC	Antibody–drug conjugate
BCMA	B-cell maturation antigen
BsAb	Bispecific antibody
CAR-T	Chimeric antigen receptor T cell
CPC	Circulating plasma cell
CR	Complete remission
GIMEMA	Gruppo Italiano Malattle Ematologiche d’Adulto
FCM	Flow cytometry
IFM	Intergroupe Francophone du Mye′ lome
SCT	Hematopoietic stem cell transplantation
MM	Multiple myeloma
MRD	Minimal residual disease
OS	Overall survival
pPCL	Primary plasma cell leukemia
PFS	Progression-free survival
RIC	Reduced-intensity conditioning
TE	Transplantation-eligible
TIE	Transplantation-ineligible
